# Fabricating a 3D floating porous PDMS − Ag/AgBr decorated g-C_3_N_4_ nanocomposite sponge as a re-usable visible light photocatalyst

**DOI:** 10.1038/s41598-024-54500-3

**Published:** 2024-02-20

**Authors:** Mohamed Taha, A. Khalid, Maryam G. Elmahgary, Shymaa S. Medany, Yasser A. Attia

**Affiliations:** 1 National Institute of Laser Enhanced Sciences, Cairo University, Giza, 12613 Egypt; 2 Department of Basic Engineering Sciences, Faculty of Engineering (Shoubra), Benha University, Benha, Egypt; 3 Department of Chemical Engineering, Massachusetts Institute of Technology, 77 Massachusetts Avenue, Cambridge, MA 02139 USA; 4 Chemical Engineering Department, The British University in Egypt (BUE), Elshrouk City, Cairo, Egypt; 5 Chemistry Department, Faculty of Science, Cairo University, Giza, 12613 Egypt

**Keywords:** Materials science, Nanoscience and technology

## Abstract

In this study, polymeric graphitic carbon nitride (g-C_3_N_4_) semiconductors was synthesized via a thermal condensation method. Subsequently, Ag/AgBr nanoparticles with varying ratios were decorated onto the g-C_3_N_4_ surface using the water/oil emulsion method. The resulting nanocomposites were characterized using XRD for phase identification and structural analysis, HR-TEM and SEM&EDAX for morphological structure, particle size, and elemental composition analysis, and XPS for investigating the chemical state and electronic structure. The impact of Ag/AgBr content on the optical properties of g-C_3_N_4_ were also studied such as (optical bandgap (E_g_), refractive index (n), extinction coefficient (k), optical conductivity (σ_opt_) and dielectric function (ε*)), Electrochemical impedance spectroscopy (EIS), PL spectroscopy and Chrono-amperometric investigations were conducted to assess the charge transfer capabilities and long-term durability of the prepared nanocomposites. The results revealed a reduction in Ag/AgBr particle size with an increase in g-C_3_N_4_ content, accompanied by a decrease in the optical bandgap from 2.444 eV to 2.393 eV. Furthermore, the nanocomposites exhibited enhanced degradation efficiencies of RhB dye, with the highest tested content of Ag/AgBr achieving 100% degradation after 120 min of irradiation. However, the challenge of catalyst separation after the degradation process remained. To address this issue, we developed a novel approach by impregnating Ag/AgBr@g-C_3_N_4_ photocatalyst onto a floating porous sponge using a simple sugar-template technique, offering potential as a reusable photocatalyst material. Furthermore, the 3D PDMS − Ag/AgBr@g-C_3_N_4_ photocatalyst was evaluated and found to maintain nearly the same photocatalytic efficiency for up to 5 consecutive cycles.

## Introduction

The main twenty-first century’s challenges are energy shortage, environmental pollution and Climate change^1^. Semiconductor Photocatalysts are widely used for photocatalysis process to convert solar energy and remove environmental pollutants. Thus, developing efficient semiconductor photocatalysts comprising low-cost metals and their compounds with high photocatalytic activity and favorable recyclability is extremely important^2–5^. Recently, graphitic carbon nitride (g-C_3_N_4_) is a promising semiconductor material due to its unique features such as; high chemical and thermal stability with 2.7 eV optical band gap value in addition to, photocatalytic activity for removal of organic pollutants from waste water and hydrogen production through water splitting^6,7^. g-C_3_N_4_ is a promising photocatalyst for solar energy conversion applications due to its appropriate electronic band structure, such as photo-electrochemical cells^8^. Additionally, g-C_3_N_4_ can be prepared through simple thermal condensation technique of different starting materials, such as melamine, urea, cyanamide and dicyandiamide^9^. The high recombination rate of photo-generated electron–hole pair minimizes its photocatalytic performance consequently, limited g-C_3_N_4_ practical applications. Therefore, various approaches are underway to enhance its optical properties through controlling morphological structure^10–12^, elemental doping of g-C_3_N_4_ may lead to the blue shift of absorption (such as sulfur doping^13^ and protonation^14^) or red shift (doping with boron, and potassium^15^), and heterostructure formation by conjugation with other semiconductor^16–19^. latterly, many studies demonstrated that plasmonic silver nanoparticles (AgNPs) can be triggered via visible light so, It is incorporated into a photocatalyst system to be utilized for energy conversion applications^20^. Meanwhile, the combination of silver halide with semiconductor photocatalyst systems can improve the electron–hole separation. Furthermore, ternary photocatalyst system based on Ag/AgX (X = halides) have enhanced the photocatalytic efficiency and stability of single photocatalyst^21–26^. For example, heterostructure photocatalysts based Ag/AgX show high optical absorption efficiency due to the synergistic effect of AgX and surface Plasmon resonance (SPR) effect of silver nanoparticles AgNPs^22,26,27^ Ag/AgCl^28^, and Ag/AgCl@TiO_2_ nanotube arrays^29^ demonstrated high efficiency toward organic dyes degradation under visible light illumination. Recently, floating porous polydimethylsiloxane (PDMS) sponge based-photocatalyst has attracted the attention of researcher due to biocompatibility, chemical stability and hydrophobic character^30^. Its hydrophobicity facilitates absorbing hydrophobic organic pollutants from water^31,32^. It can be easily fabricated through a sugar cube template method without using any hazardous chemicals^33^. Recent studies on the PDMS-based photocatalyst sponge have shown some promising results^34–36^.

In this work, ternary photocatalyst composed of g-C_3_N_4_ with Ag/AgBr were prepared via emulsification method with different ratios, and evaluate their crystal structure, microstructural parameters by XRD analysis, Morphology, size & elemental composition via (HRTEM and SEM&EDAX) and the chemical state and electronic structure by XPS technique. Additionally, The optical parameters were also studied such as (optical bandgap (Eg), refractive index (n), extinction coefficient (k), optical conductivity σopt and dielectric function (ε*)), Electrochemical impedance spectroscopy (EIS) and Chrono-amperometric investigations were conducted and the photocatalytic activity was evaluated by RhB degradation under visible light irradiation. Finally, a porous plasmonic floating photocatalyst based on PDMS − Ag/AgBr@g-C_3_N_4_ composite sponge was fabricated to form flexible substrate allows the reuse of the photocatalyst several times.

## Experimental methods

### Materials

2,4,6-Triamino-1,3,5-triazine 99% (Melamine) was obtained from Across organics™, silver nitrate (AgNO_3_) ACS reagent ≥ 99.0%, Absolute ethanol ACS, ISO, Reag, isopropanol 99.9%, Acetone HPLC grad and chloroform ACS, ISO, Reag. were obtained from Merck Millipore™-Germany, N-Cetyl-N, N, N, trimethyl ammonium bromide AR (CTAB) was supplied from Loba-Chemie™-India. Perfluorinated resin solution containing Nafion™ 1100W and SYLGARD® 184 were supplied from Sigma-Aldrich. All materials were used without further purification. Solutions were prepared using double distilled water (DDH_2_O).

### Preparation of pure g-C_3_N_4_

**Figure 1:**
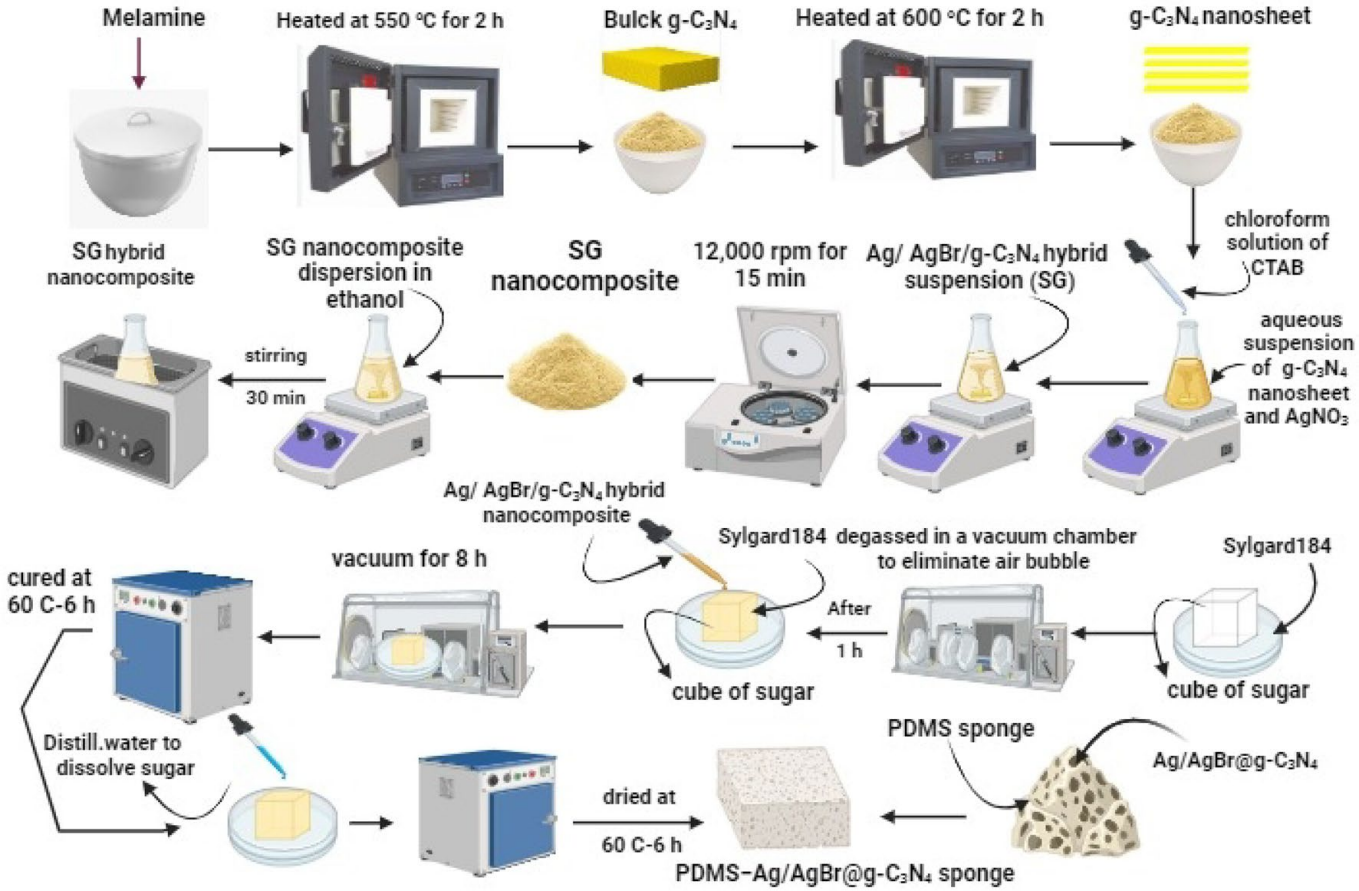
The schematic diagram for the fabrication process.

g-C_3_N_4_ was prepared via Thermal condensation technique with little modification (Fig. 1)^7,37,38^. Briefly, 10 gm melamine powder heated at 550 °C for 2 h in closed system with heating rate of 5 °C /min after that, the formed g-C_3_N_4_ was crushed and exfoliated thermally by heating in open air at 600 °C for 2 h and excessive ammonia has been removed.

### Preparation of ternary photocatalyst (Ag/AgBr@g-C_3_N_4_)

AgBr@g-C_3_N_4_ heterostructure was synthesized via a modified water/oil emulsion method (Fig. 1)^7,39,40^. Typically, 0.4 mL aqueous suspension of g-C_3_N_4_ with different concentration (1, 5, and 10 mg ml^−1^) (SG1, SG2, and SG3) respectively and a 0.5 mL aqueous solution of AgNO_3_ (0.1 M) were added dropwise into a 10 mL chloroform solution of CTAB (8.23 × 10^–3^ mol L^−1^) under vigorous stirring which maintained for another 30 min, after which a off-white suspension containing AgBr/g-C_3_N_4_ hybrid nanocomposites was obtained and signed as SG1, SG2, and SG3 respectively. The resulting white yellowish precipitate were collected by centrifugation at 12,000 rpm for 15 min (Hermle Z32 HK, Germany) and washed multiple times with ethanol then with double distilled water by the same method.

For pure Ag/AgBr NPs preparation, the previous steps were repeated in absence of g-C_3_N_4_.

### Physico-chemical characterization of the prepared materials

The crystalline structure and phase identification of the synthesized materials was investigated using Empyrean Malverpanalytical, Netherland X- ray diffraction, with 2-Theta (5.0°—85°), with step size 2-Theta: 0.04 and at (Kα) = 1.54060°. the morphological structures were performed via high resolution transmission electron microscope (HRTEM, JEOL JEM-2100 at an accelerating voltage of 200 kV) and Field emission Scanning electron microscope (FESEM, Quattro S, Thermo Scientific), In addition, the elemental composition of each sample confirmed with Energy dispersive X-ray (EDAX) and Mapping. The chemical state and electronic structure of photocatalysts were determined by using X-ray photoelectron spectroscopy (XPS, ESCALAB 250Xi, Thermo Fisher, USA). The UV–vis diffused reflectance spectra (UV–Vis-DRS) were determined via V-570 UV–Vis spectrophotometer JASCO Corp., in a range of 200–800 nm. Photoluminescence (PL) measurements were conducted at room temperature with laser wave length 360 nm via Lumina Fluorescence Spectrometer- Thermo-Scientific.

### Fabrication of PDMS sponge and PDMS − Ag/AgBr@g-C_3_N_4_ sponge

Sacrificial sugar cube template method used to fabricate a PDMS sponge as illustrated in Fig. 1^41^. Briefly, a 10:1 ratio mixture of PDMS prepolymer (Sylgard 184 A, 15 g) and curing agent (Sylgard 184 B, 1.5 g) was placed in a glass Petri dish and degassed in a vacuum chamber for 1h to eliminate any air bubbles. To fabricate a PDMS − Ag/AgBr@g-C_3_N_4_ sponge, first, Ag/AgBr@g-C_3_N_4_ was dispersed in anhydrous ethanol to make a 10% (w/v) suspension and sonicated for 30 min. Then, 2.5 mL was injected portion by portion into the sugar cube to decorate porous interfaces of the sugar cube with Ag/AgBr@g-C_3_N_4_. This sugar cube contains Ag/AgBr@g-C_3_N_4_ was used to absorb the PDMS precursor under vacuum for 8 h through a capillary action to make a PDMS − Ag/AgBr@g-C_3_N_4_ sponge. The filled sugar cube was placed in a convection oven at 60 °C and cured for 6 h. After curing, the sugar templet was removed by dissolving in water, leaving a PDMS − Ag/AgBr@g-C_3_N_4_ porous sponge. Then, dry the sponge in the convection oven at 60 °C for 12h.

### The photocatalytic measurement

The photocatalytic activity of the nanoparticles was evaluated under UV light irradiation using a 40 W UVA Xe-lamp (Sylvania, Germany) as the light source. The photodegradation experiments involved adding 0.1 g of the photocatalyst to 100 mL of 1 × 10^–5^ M of RhB dye. The mixture was stirred for 1 h in the dark to achieve equilibrium of adsorption–desorption then, the photocatalytic reaction is initiated by turning on the light. 5 mL sample was taken every 30 min and then centrifuged to settle the photocatalyst and decant the pure dye. A UV–vis spectrophotometer (Shanghai Metash Instruments, UV-5100) was used to measure the absorbance of the dye solution which was then correlated to its concentration using beer lambert's law.

### Recyclability test

To investigate the recyclability of PDMS-(Ag/AgBr@g-C_3_N_4_) sponge, its efficiency for RhB degradation was evaluated for five runs. The PDMS-(Ag/AgBr@g-C_3_N_4_) sponge was washed with deionized water several times between each run.

### Electrode preparation and electrochemical measurements

As the working electrode, the glassy carbon electrode with a surface area of 0.071 cm^2^ is utilized. It was then rinsed with double-distilled water DD.H_2_O and ethyl alcohol after being polished with soft emery paper. Then, catalysts were produced by ultrasonically suspending catalyst powder (15 mg) in a mixture of 0.5 mL isopropanol, 0.5 mL DD.H_2_O, and 0.5 mL 5 wt.% Nafion solutions for 15 min. After this, 50 μL of ink was applied to the surface of the glassy carbon electrode and left to dry overnight in a desiccator. Autolab PGSTAT128N was utilized to collect data for electrochemical impedance spectroscopy (EIS), and chronoamperometry using NOVA electrochemistry program, the impedance spectrum was fitted (Version 2.1, Metrohm Autolab, Utrecht, Netherlands). Three electrode cells were built using Ag/AgBr, SG1, SG2, SG3 and g-C_3_N_4_ catalysts as working electrodes, Ag/AgCl /KCl (sat.) and Pt wire as a reference electrode and an auxiliary electrode respectively. A consistent AC voltage value was maintained during EIS tests by applying an AC voltage amplitude of 10 mV and a frequency range of 1 × 10^4^ Hz to 100 mHz. All electrochemical experiments were conducted in de-aerated solutions at ambient temperature.

## Results and discussion

### Crystal structure and morphology of g-C_3_N_4_, Ag/AgBr and nanocomposites

**Figure 2:**
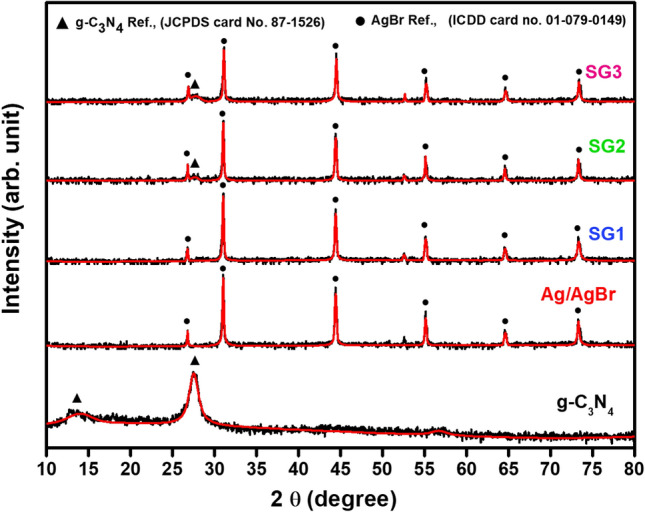
Shows the XRD pattern of the prepared materials g-C_3_N_4_, Ag/AgBr, SG1, SG2 and SG3.

XRD patterns were performed to influence the phase identification and structural characteristics of the prepared samples. The phase identification and the line profile analysis were performed using the High score plus software. The obtained patterns for g-C_3_N_4_, Ag/AgBr and their nanocomposites are shown in Fig. 2. For g-C_3_N_4_ the peak at 27.36° is the characteristic peak for (002) orthorhombic plane assigned to the stacking of conjugated aromatic planes in g-C_3_N_4_^42^ meanwhile, the peak at 13.7° attributed to (100) hexagonal plane of g-C_3_N_4_, which represented an in-plane structural packing motif with a period of 0.675 nm (JCPDS card No. 87–1526)^43,44^. All the diffraction peaks for the pure Ag/AgBr and SG1, SG2, and SG3 samples were matched to the polycrystalline AgBr phase with a Cubic Face centered structure of space group *Fm-3m* (ICDD card no.01–079-0149). Briefly, the peaks at 26.77°, 31°, 44.39°, 52.55°, 55.1°, 64.55°, 73.29°, and 81.65° are related to the (111), (200), (220), (311), (222), (400), (420), and (422) planes of Ag/AgBr. Upon adding a different ratio of the g-C_3_N_4_, an additional peak at 27.5^o^ was observed and assigned to the g-C_3_N_4_ phase. No other foreign peaks were observed, verifying the purity of the samples. Moreover, the intensity of this peak became more intense and resolved with increasing the ratio of g-C_3_N_4_. Thus, the prepared composites consisted of only two phases, Ag/AgBr and g-C_3_N_4_.

The Crystallite sizes (D), the internal micro-strain (ε), and dislocation density (δ), for the principal diffraction peak at 2θ ≃ 31°, were estimated from the line profile analysis via the following relations.

1 $$D_{{\left( {200} \right)}} = \frac{K \lambda }{{\beta_{{\left( {200} \right)}} cos\theta }}$$

2 $$\varepsilon_{{\left( {200} \right)}} = \frac{{\beta_{{\left( {200} \right)}} }}{4\tan \theta }$$

3 $$\delta_{{\left( {200} \right)}} = \frac{1}{{D_{{\left( {200} \right)}}^{2} }}$$

where *k* is a constant, λ is the applied wavelength (1.540 Å), and β is the integral breadth. The results, which have been obtained and are presented in Table 1, exhibit a clear indication of the reliance of the microstructural parameters on the content of g-C_3_N_4_. Upon the introduction of g-C_3_N_4_ (SG1), there is a slight reduction in the crystallite size, accompanied by an increase in microstrain, and hence the dislocation density. By elevating the proportion of g-C_3_N_4_ (SG2), the crystallite size continues to decrease, resulting in a further increase in both microstrain and dislocation density. Subsequent to this, if the content of g-C_3_N_4_ (SG3) is increased, there is a marked reduction in the crystallite size and an increase in the dislocation density.

**Table 1:** Summarize the XRD parameters of Ag/AgBr, SG1, SG2 and SG3.

Sample ID	2*θ* (°)	Integral breadth	Crystallite size (nm)	Strain % (ε)	Dis. Density (δ)
Ag/AgBr	31.00	0.24656	33.4	0.3881	8.929E−6
SG 1	30.99	0.28505	28.9	0.4483	1.195E−5
SG 2	31.07	0.28985	28.4	0.4552	1.235E−5
SG 3	30.99	0.29911	27.5	0.47041	1.316E−5

**Figure 3:**
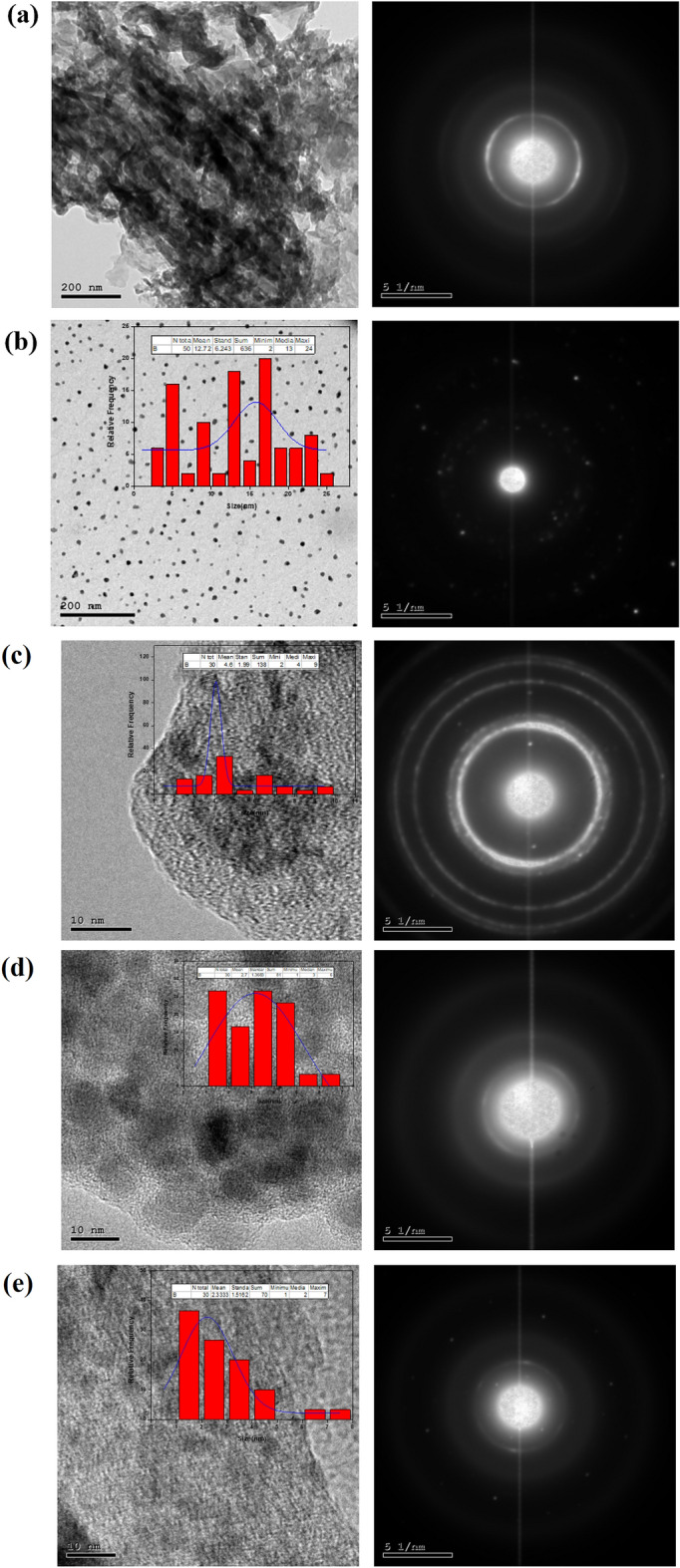
Depicts the TEM image and Selected area electron diffraction of g-C_3_N_4_ (**a**), Ag/AgBr (**b**), SG1 (**c**), SG2 (**d**), and SG3 (**e**). The inset Fig. is the Histogram of crosseponding material.

**Figure 4:**
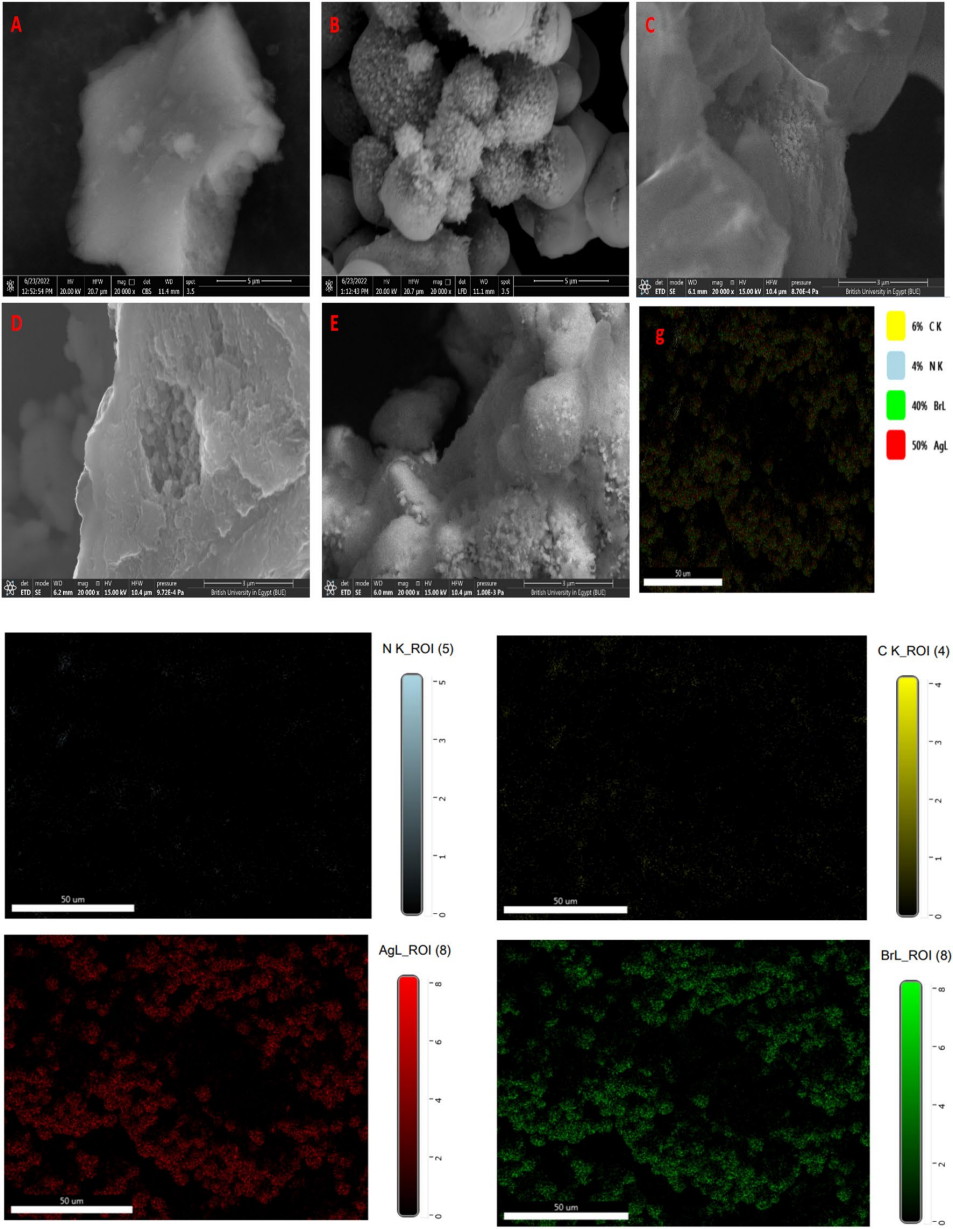
Depicts the SEM images of g-C_3_N_4_, Ag/AgBr, SG1, SG2, and SG3 (**a**, **b**, **c**, **d**, and, **e** respectively) and g). elemental mapping of SG1.

The morphological structure, SAED and corresponding histogram of g-C_3_N_4_, Ag/AgBr, SG1, SG2 and SG3 were illustrated by TEM, SEM and EDAX analysis. Briefly, the morphology structure of of g-C_3_N_4_ were displayed by TEM (Fig. 3a) and SEM (Fig. 4a), it can be shown that the g-C_3_N_4_ shows 2D- structure from wrinkled platelet structure, which is expected to provide active sites for the Ag/AgBr particles growth^45^. Figures 3b and 4b depicts the TEM and SEM images of Ag/AgBr respectively, indicated formation of 0D structure of Ag/AgBr with average particle size around 12.72 nm, the TEM image with corresponding histogram and SAED of SG1, SG2 and SG3 nanocomposites displayed in Fig. 3c–e respectiviely, its clearly observed the distribution of 0D Ag/AgBr nanoparticles over 2D g-C_3_N_4_ to form the hetero-junction in all nanocomposites as well as, confirmed by SEM images of the Composites materials. Moreover, the particle size of Ag/AgBr decreased as the content of graphitic carbon nitride (g-C_3_N_4_) increased. Specifically, the particle size decreased from 4.6 nm for SG3 to 2.7 nm and 2.33 nm for SG2 and SG1, respectively. These observations align with previous studies, supporting the notion that a higher g-C_3_N_4_ content in the nanocomposites promotes Ag/AgBr adsorption and stabilization, ultimately leading to a reduction in particle size^27,46^.

Additionally, The EDAX was utilized to investigate the elemental composition of the synthesized samples g-C_3_N_4_, Ag/AgBr, SG1, SG2, and SG3. For g-C_3_N_4_ as conducted in Table 2, for g-C_3_N_4_ Only carbon and nitrogen are present in its composition with atomic percent 43.37% and 56.63% respectively indicated pure formation of g-C_3_N_4_ while, The Ag/AgBr constituents are Ag and Br. Furthermore, EDAX spectra of SG1, SG2, and SG3 show presence of elemental C, N, Ag, and Br with variable atomic percent, in which the content of Ag/AgBr gradually increased approximately in order 80%, 50%, and 40% w/w for SG1, SG2, and SG3 respectively which indicates that the formation of heterojunction between g-C_3_N_4_ and Ag/AgBr photocatalyst. Figure 4 displayed the elemental mapping of SG1 nanocomposite indicated homogenous distribution of Ag/AgBr nanoparticles over g-C_3_N_4_.

**Table 2:** The elemental analysis of g-C_3_N_4_, Ag/AgBr, SG1, SG2, and SG3 respectively by EDAX.

Catalyst	N		C		Ag		Br	
	Weight %	Atomic %	Weight %	Atomic %	Weight %	Atomic %	Weight %	Atomic %
g-C_3_N_4_	60.36	56.63	39.64	43.37	–	–	–	–
Ag/AgBr	–	–	–	–	55.15	47.67	44.85	52.33
SG1	14.95	37.9	11.5	34.01	39.82	13.11	33.73	14.99
SG2	31.04	49.86	20.6	38.59	28.27	5.9	20.1	5.66
SG3	37.02	51.66	24.78	40.31	20.72	3.75	17.49	4.28

### XPS analysis

**Figure 5:**
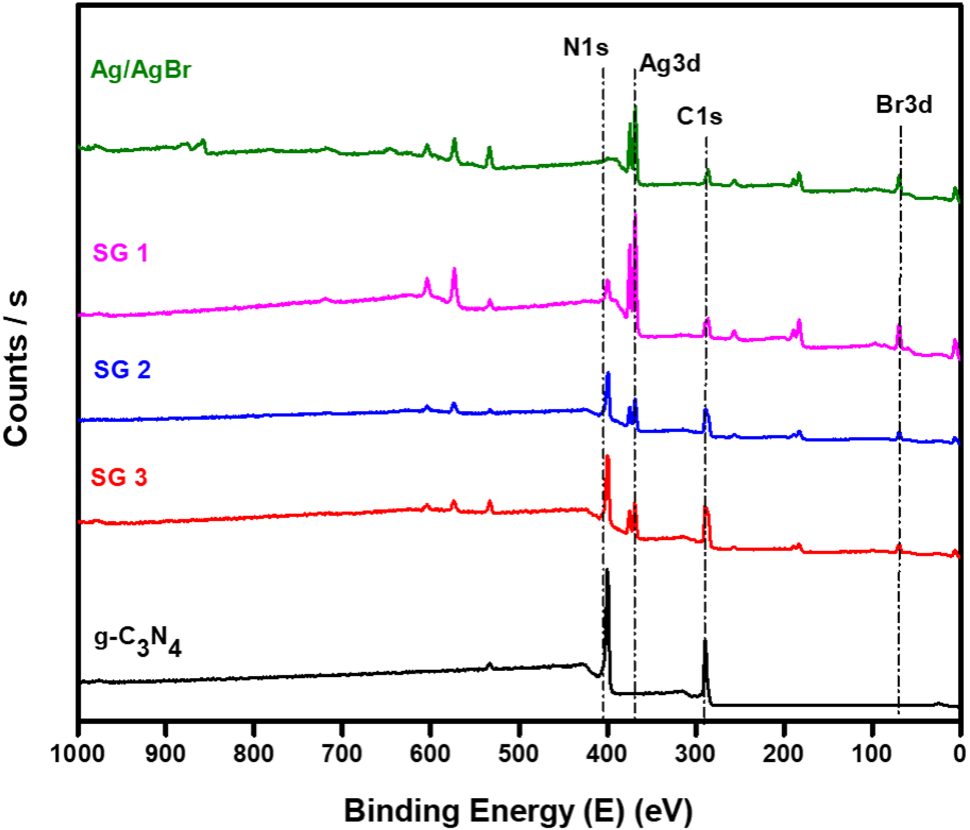
Shows the fully scanned XPS spectra of g-C_3_N_4_, Ag/AgBr, SG1, SG2, and SG3.

The chemical state and electronic structure of the as-prepared g-C_3_N_4_, Ag/AgBr, SG1, SG2, and SG3 were performed using XPS, and the results of the full scan spectra of the g-C_3_N_4_, Ag/AgBr, SG1, SG2, and SG3 materials in the range of 0–1000 eV are presented in Fig. 5. The spectrum of pure g-C_3_N_4_ shows that C, N, and O exist while, the pristine Ag/AgBr spectrum contains Ag, Br, C and O elements. Furthermore, C, N, Ag, Br, and O coexist in the all nanocomposites (SG1, SG2, and SG3) spectra. The appearance of O attributed to molecular oxygen adsorption onto the materials surface.

**Figure 6:**
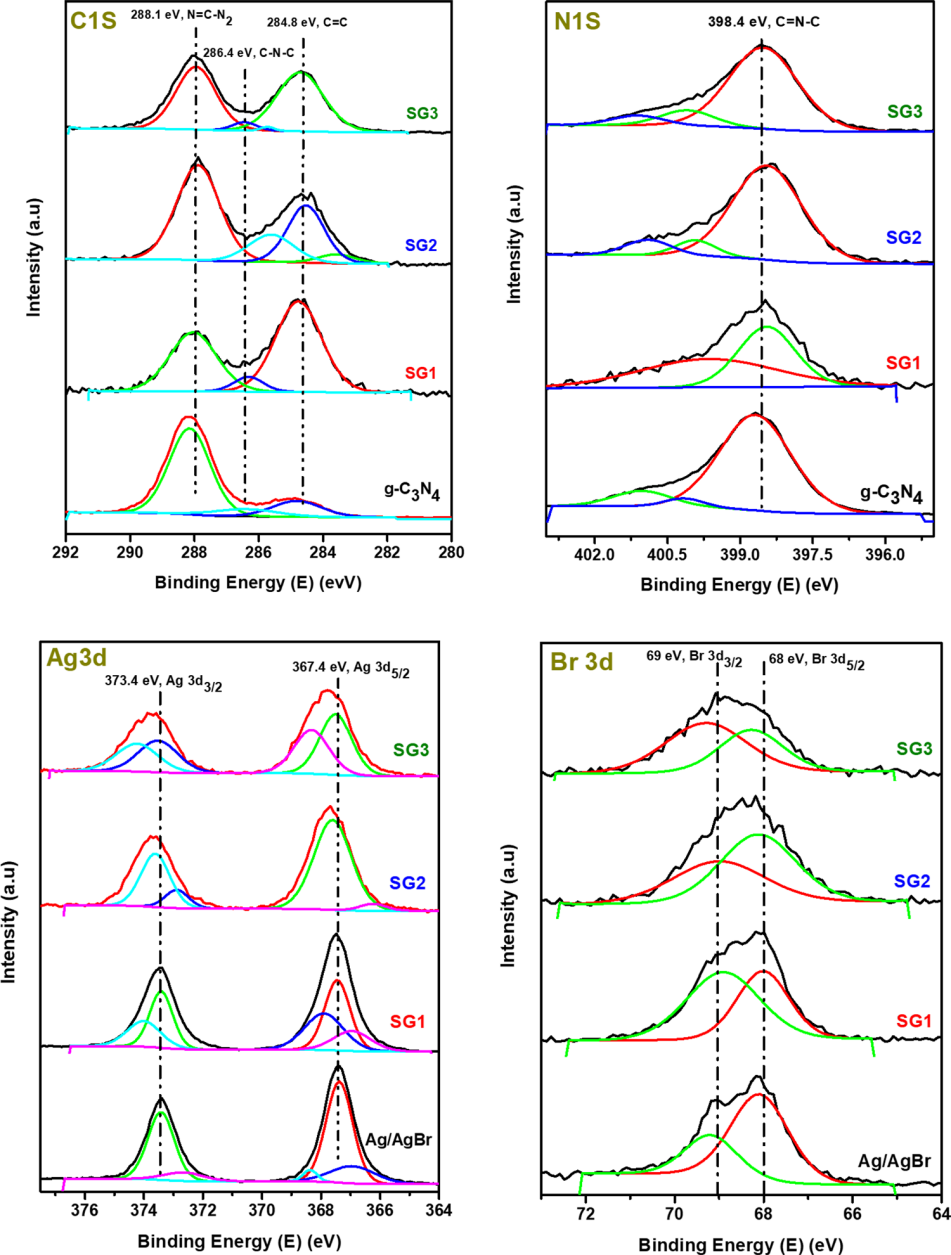
Depicts the high resolution XPS spectra (C 1*s*, N 1*s*, Ag 3*d* and Br 3*d*) for as prepared materials g-C_3_N_4_, Ag/AgBr, SG1, SG2 and SG3.

The C 1*s* high-resolution spectra is seen in Fig. 6C fitted into three characteristic peaks at 284.8, 286.4 and 288.1 eV, which correspond to the binding energy of sp^2^ C = C band, sp^2^ -hybridized carbon in the C–N– C and tertiary carbon N = C-N_2_, in g-C_3_N_4_ structure, respectively^45,47,48^. The main N 1s peak at 398.4 eV assigned to *sp*^2^ -hybridized aromatic N bound to C atoms (C = N–C; Fig. 6 N 1*s*) while, the peak at 399 eV is assigned to either tertiary nitrogen of motif structure C_6_N_7_ or amino groups carrying hydrogen ((C)2NH) in connection with structural defects due to incomplete thermal condensation. The peak at 401 eV is from the N–H structure. The small peak at 403.9 eV is assigned to π excitation^45,48^. It is clear that the N 1*s* in the SG1, SG2, and SG3 composites shifts slightly from 398.4 eV for pristine g-C_3_N_4_ to 398.45, 398.45, 398.44, and 398.5 for SG1,SG2, and SG3 nanocomposites respectively, which indicate that the chemical environment of N in the composites has changed due to the interaction between Ag^+^ of AgBr and lone pair of electron of nitrogen of g-C_3_N_4_^49^.

The high-resolution Ag 3*d* XPS spectra are shown in Fig. 6 Ag 3*d*, and the Ag 3*d* spectrum of Ag/AgBr composed of two separated peaks at 367.42 assigned to binding energy of Ag 3*d*3/2 and 373.42 eV, which is assigned to binding energy Ag 3*d*5/2 corresponding to Ag^+^ of AgBr^50,51^. Furthermore, the peak of Ag 3*d*_3/2_ is fitted into two peaks at 367.14 and 368.52 eV suggested to existence of Ag^+^ and Ag^0^ to form Ag/AgBr^51^. Additionally, the peak of Ag 3*d*_5/2_ is split into two peaks at 372.66 and 373.82 eV^52–54^. The peak around 368 eV is assigned to silver metal Ag^0^ and since g-C_3_N_4_ facilitates the reduction of metal cation^44^, its intensity increased as g-C_3_N_4_ increased. While, the peaks at 367.2 and 373.4 eV are assigned to silver ion Ag^+^ of AgBr. The XPS spectra of Ag 3*d* and N 1*s* confirm the existence of traces amount of Ag metal species and the interaction between Ag/AgBr and g-C_3_N_4_ to form heterostructure between both of them. Two bands at approximately 68 eV and 69 eV ascribed to Br 3*d*_5/2_ and Br 3*d*_3/2_, respectively, are observed for pure Ag/AgBr and nanocomposites in Fig. 6, which is matched with those of Ag/AgBr^49^.

### Optical properties

**Figure 7:**
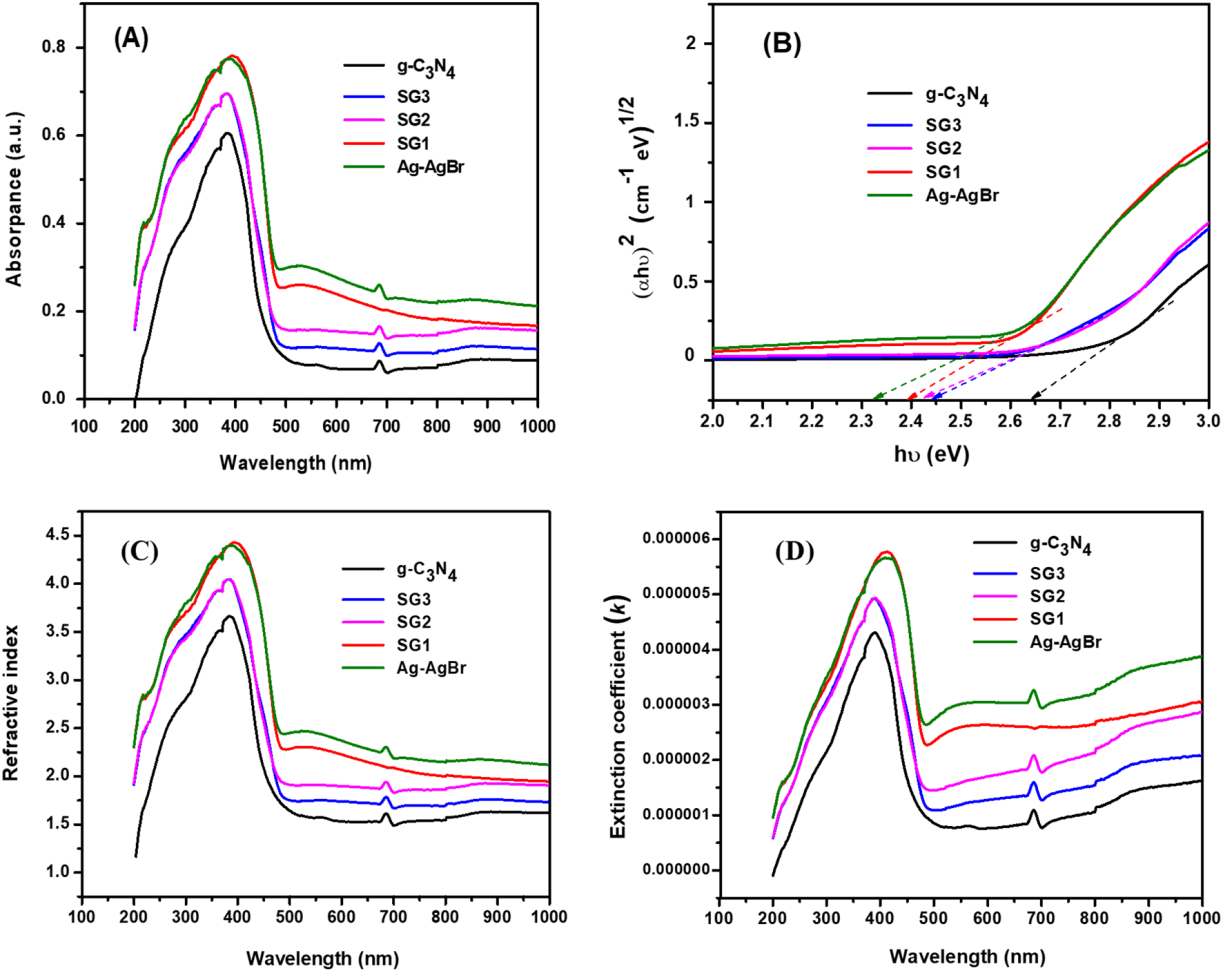
Shows the (**a**) Variation of the absorbance as a function of wavelength, (**b**) Tauc's plot, Variation of (**c**) Refractive index and (**d**) Extinction coefficient as a function of wavelength for g-C_3_N_4_, SG1, SG2, SG3, and Ag/AgBr.

The optical properties of g-C_3_N_4_, SG1, SG2, SG3, and Ag/AgBr were determined via UV–Vis absorption. As shown in Fig. 7.a, pure g-C_3_N_4_ displayed a typical semiconductor absorption in the range of 200–500 nm with maximum absorption edge at 384 nm, which was attributed to the electronic excitation from valence band to conduction band^43,55^. Pure Ag/AgBr displayed a strong absorption in all tested region^56,57^. Furthermore, the spectra of heterostructures with different Ag/AgBr content (SG1, SG2, and SG3) were shown in Fig. 7a. The absorption thresholds of SG1, SG2, and SG3 demonstrated a negligible shift (small slight red shift) in addition, the intensity of optical absorption was improved in order to increase content of Ag/AgBr, which was attributed to synergistic effect of AgBr/g-C_3_N_4_ interface and SPR effect of silver nanoparticles AgNPs^22,26,58^.

The essential parameter of the optical absorption is the electronic transitions which, controlled by certain selection rules, which can be expressed according to Tauc's relationship^59^.

4 $$\mathrm{\alpha h\upsilon }={\text{A}}({\mathrm{h\upsilon }-{{\text{E}}}_{{\text{g}}})}^{{\text{n}}}$$

where *hʋ* is the photon energy,* α* is the absorption coefficient, *A* is a constant, *h* is constant of Planck and *k* is a constant with allowed values of 1/2 and 2 and not allowed values of 3/2 and 3 for direct and indirect transitions respectively. The n value of direct semiconductor is 4 while for indirect semiconductor is 1. g-C_3_N_4_ regard as a direct semiconductor, and AgBr is indirect semiconductor, so their n values are 4 and 1, respectively^60,61^. The value of band-gap is calculated by the Tauc-plot curve of (*αhʋ)*^*1/2*^ value versus (*hʋ*) as shown in Fig. 7b. the optical band-gap values of materials g-C_3_N_4_, Ag/AgBr, SG1, SG2, and SG3 were calculated based on Tauc-plot from the energy axis intercept. The estimated band gaps are 2.646 eV, 2.444eV, 2.425 eV ,2.393 eV, and 2.325 eV, for g-C_3_N_4_, SG3, SG2, SG1, and Ag/AgBr.

The values of optical bandgap slightly decrease as the Ag/AgBr concentration increases. In other words, an increase in the content of Ag/AgBr leads to a shift of the absorption edge towards lower values of bandgap, form 2.646 eV (pure g-C_3_N_4_) to 2.393 eV (SG1). This is attributed to quantum confinement phenomena and to SPR effect of AgNPs and the good photosensitive ability of AgBr^26,57^.

The index of refraction, n, is crucial for examining a material's optical qualities. The refractive index of materials is fascinating because of its relationship with the local electric field and electronic polarizability. It also determines the materials' compatibility with optoelectronic devices. Based on the reflection (R) and attenuation coefficient (k), the refractive index was computed as follows^60^,

5 $$n = \left[ {\frac{1 + R}{{1 - R}}} \right] + \sqrt {\left[ {\frac{4R}{{(1 - R)^{2} }}} \right] - k^{2} }$$

6 $$k = \frac{\alpha \lambda }{{4\pi }}$$

where *α* is the coefficient of absorption. Figure 7c, d illustrate variability of n and k in relation to the incident light’s wavelength for g-C_3_N_4_, SG1, SG2, SG3, and Ag/AgBr respectively.

The refractive index increases as wavelength increases until it reaches its maximum value before going down, as seen in Fig. 7c. This maximum point slightly redshifted upon increase the content of Ag/AgBr conjugated with g-C_3_N_4_. The correlation between the incident wavelength and plasma frequency in the strong absorption area may explain an increase in refractive index, whereas the correlation between normal light dispersion and a decreasing in absorption may explain the decrease in refractive index^62,63^.

The variation in the value of extinction coefficient k as a function of wavelength is displayed in Fig. 7d. the extinction coefficient is the loss of light caused by absorption and scattering of light per unit distance in a testing substance. It is noted as the behavior of the k graph is similar to that of the refractive index except that after maximum point, it decreases and almost constant at 700 nm > λ > 500 nm and then λ > 500 nm increase slowly. Moreover, the *k* value for SG1 is higher than that for g-C_3_N_4_, SG2, SG3, and Ag/AgBr. This may be a sign that the loss of energy to absorption from the material has increased due to high efficiency of optical absorption of SG1.

#### Optical dielectric constants and optical conductivity

The complex dielectric function (ε*) is a sum of the real part (The optical dielectric constant ε′) and the imaginary part (the optical dielectric loss ε″). The two parties of complex dielectric function depended on the refractive index (n) and extinction coefficient (k) according to the following relations^60,62^.

7 $$\varepsilon^{\prime } = \, n^{2} {-} \, k^{2}$$

8 $$\varepsilon^{\prime \prime } = { 2}n \, k$$

**Figure 8:**
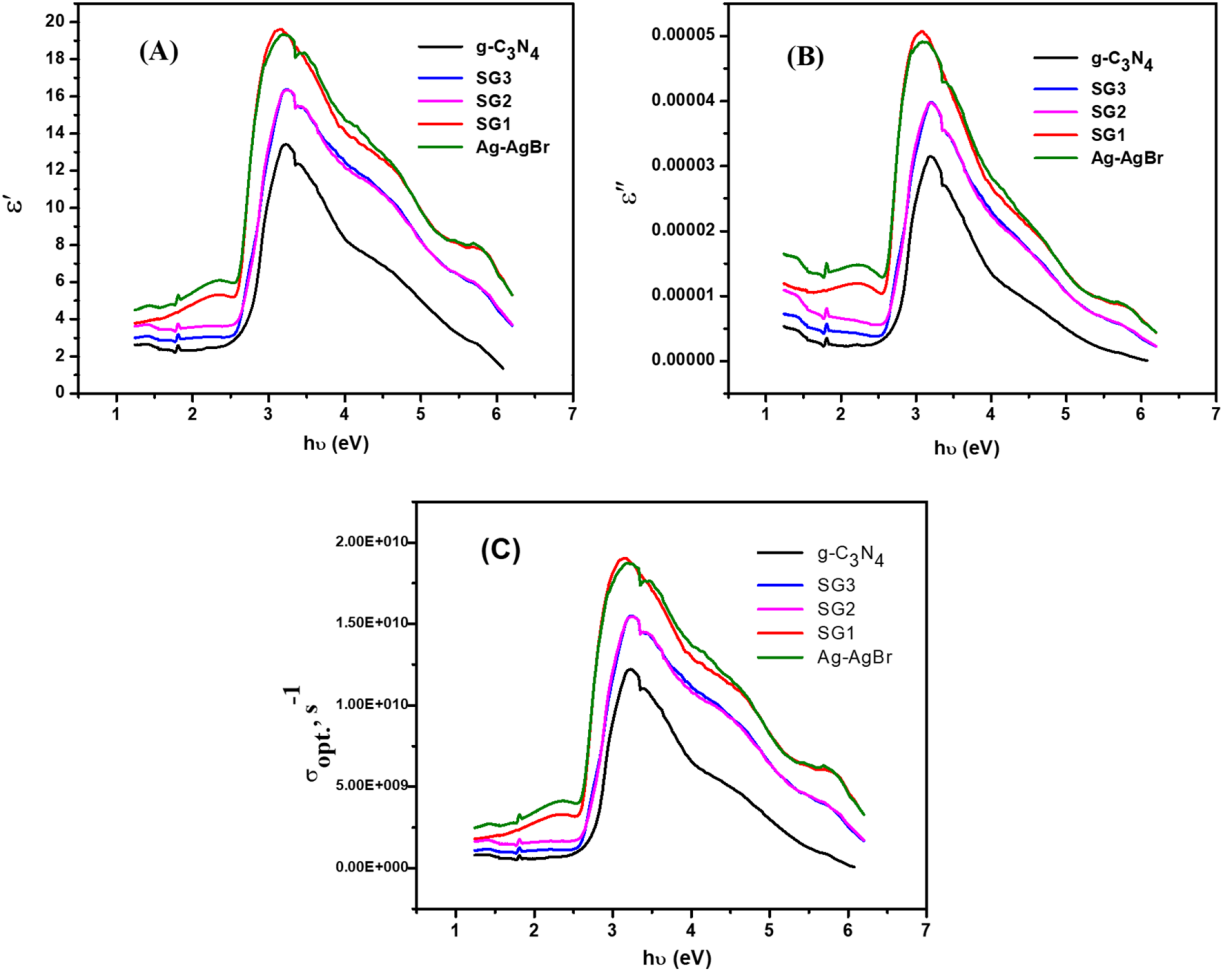
Variation of (**a**) ε′, (**b**) ε″ as a function of photon energy and (**c**) the variation of the optical conductivity versus the photon energy for prepared samples.

The dependence of the real part (ε′) and the imaginary part (ε″) of complex dielectric function on the photon energy for all the studied samples are illustrated in Fig. 8a,b, respectively.

It is noted that ε′ and ε″ values have maximum in the range from ∼2.8 eV to 4.3 eV. This was a result of augmentation. absorption of Ag/AgBr in Ag/AgBr/g-C_3_N_4_ nanocomposite. Moreover, these values augmentee as the Ag/AgBr concentration increases. Adding Ag/AgBr to g-C_3_N_4_ causes a small shift and augmentee in the ε′ and ε″ values, which can be attributed to incident photon-free electron (photo-generated electron) interactions in the nanocomposites^64^

The optical conductivity σ_opt._ of such materials depends on its optical bandgap, absorption coefficient, extinction coefficient, frequency of incident photons and the refractive index. It was calculated by the following equation^60,62^**.**

9 $${\sigma _{{\text{opt}}}} = \alpha \,{\text{n}}\,{\text{c}}/4\pi$$

where *c* is the light speed. Figure 8c depicts the relationship between the optical conductivity and photon energy for all the studied samples. It is showed that the optical conductivity increased until maximum in the range ∼3–4 eV then decreases up to 6 eV. This is because of the increase in absorption coefficient and obtaining the maximum absorption in the same region^59,62^. Therefore, for the maximum absorption coefficient of the material (as in Fig. 8c), the amount of free charge carriers excited from the valance band to conduction band grows and σ_opt_ increases as well. The σ_opt_ value for SG1 is higher than that for g-C_3_N_4_, SG2, SG3, and Ag/AgBr. Because of the higher absorbent nature of SG1 the photo-generated carriers were increased hence, the optical conductivity σ_opt_ enhanced indicating highly efficient of electron–hole separation in SG1 nanocomposite^65^.

### Electrochemical measurements and PL spectroscopy

**Figure 9:**
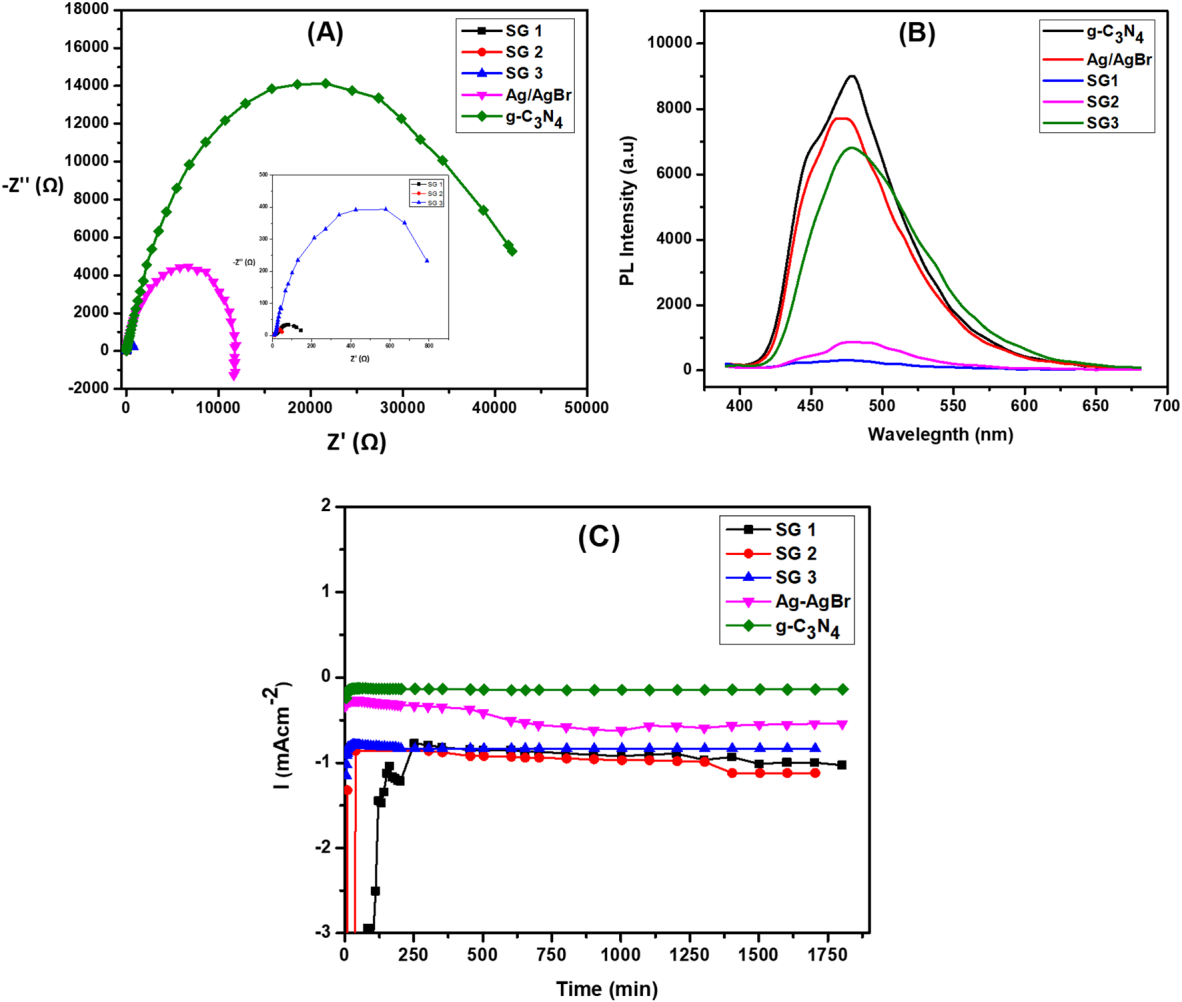
(**a**) Nyquist plots of prepared samples g-C_3_N_4_, Ag/AgBr, SG1, SG2, and SG3 at constant potential (0.6 V) in 0.1 M H_2_SO_4_ solution_._ (**b**) PL spectra of g-C_3_N_4_, Ag/AgBr, SG1, SG2, and SG3 and (**c**) Chronoamperograms of different electrodes g-C_3_N_4_, Ag/AgBr, SG1, SG2, and SG3 in 0.1M H_2_SO_4_ at constant potential + 0.6 V for 5h.

Electrochemical impedance spectroscopy (EIS) tests were performed on a variety of electrodes to examine the impact of modifying the surface composite on the electrochemical behavior and charge transfer capabilities. Figure 9a showed Nyquist plot of g-C_3_N_4_, Ag/AgBr, SG1, SG2, and SG3 in 0.1M H_2_SO_4_ solution potential of + 0.6 V. The electrode with the highest catalytic activity SG1, has the lowest resistance for the charge transfer, indicated high separation of photo-generated charge hence, high catalytic activity. In contrast, the g-C_3_N_4_ has high charge transfer resistance indicated the most insufficient activity of photo-generated electron charges. Additionally, photoluminescence (PL) technique is used to confirm the transfer of photo-generated carriers in a photocatalyst. As Fig. 9b illustrates, all tested materials were evaluated using an excitation wavelength of λ = 360 and their PL emission showed in the range 400–600 nm. Briefly, pristine g-C_3_N_4_ shows a highest PL emission at about 478 nm, indicating the high re-combination rate of photo-generated carriers. The PL peak of Ag/AgBr is weaker than that of g-C_3_N_4_. Upon conjugation between g-C_3_N_4_ and Ag/AgBr, the PL intensity decreased in order SG3, SG2 and SG1 respectively, indicating a significant inhibition of electron–hole pair recombination. These results are according well with the previous studies^27,48^.

Using a chronoamperometric investigation at a potential of 0.6 V in a 0.1M H_2_SO_4_, the long-term durability of electrodes made g-C_3_N_4_, Ag/AgBr, SG1, SG2 and SG3 was examined, as illustrated in Fig. 9c. Five hours were dedicated to the chronoamperometry studies. Due to the constant potential in the first few seconds, the capacitive current diminished, causing the current to decrease. As the oxidation process continues, the extension of the diffusion layer into the bulk electrolyte is responsible for the constant fall in current density value. Then, a steady state is attained. All examined photocatalyst exhibited a stable performance for prolonged reactions; however, the steady state oxidation current density value varied. In summary, the graphitic carbon nitride (g-C_3_N_4_) exhibited a stable performance, attributed to the strong pi-bonding within its carbon and nitrogen framework^66^ However, upon conjugation of g-C_3_N_4_ with Ag/AgBr, as previously discussed, the Ag^0^ content increased. This resulted in a direct enhancement of the current density in the SG samples, where the Ag^+^ species were present in an acidic medium^67^. Eventually, a steady state was achieved, indicating the successful integration and improved performance of the nanocomposite materials.

### Photocatalytic measurements

**Figure 10:**
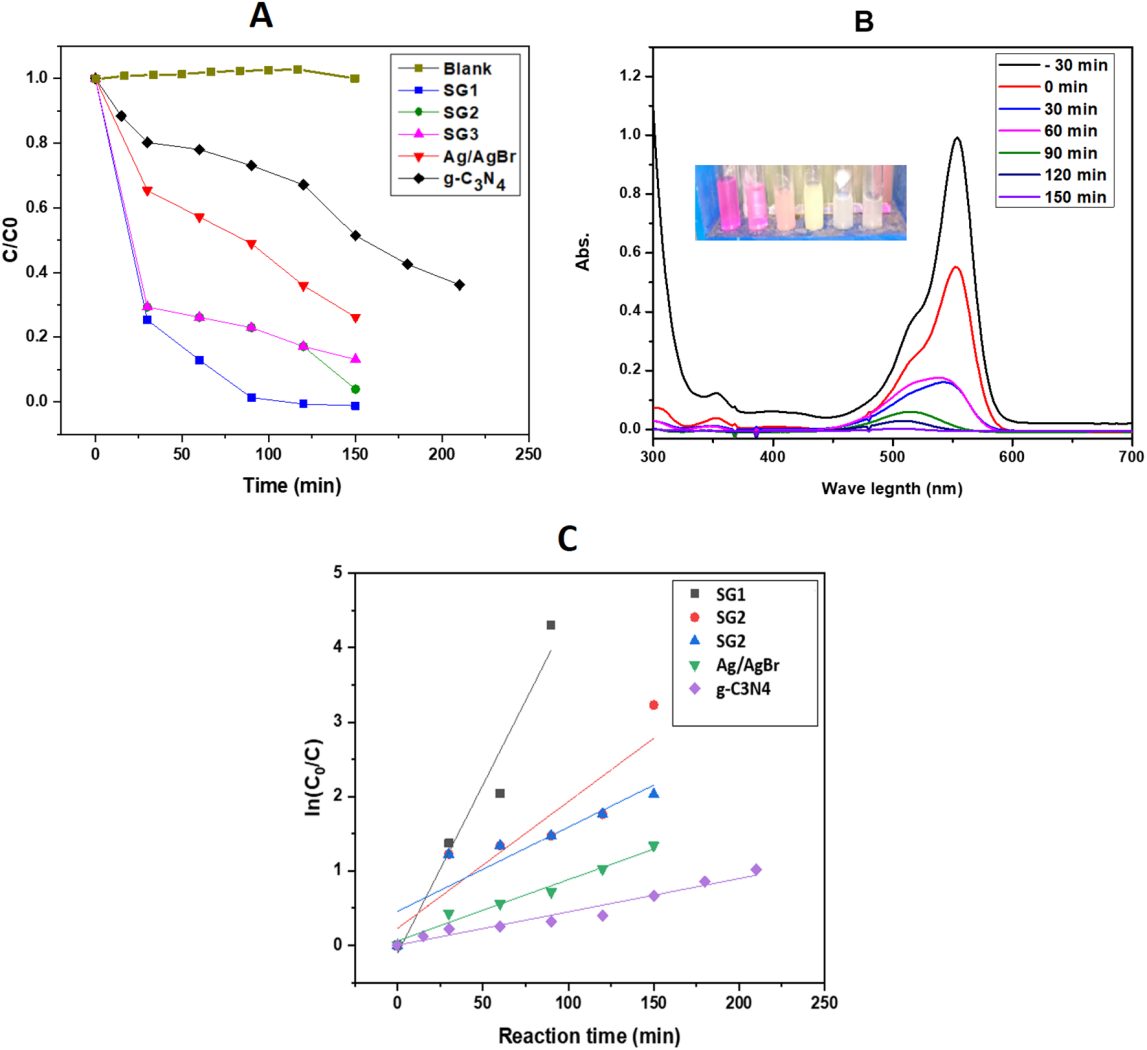
(**A**) The photocatalytic degradation of RhB with g-C_3_N_4_, Ag/AgBr, SG1, SG2, and SG3 under visible-light. (**B**) absorption spectral changes of RhB by SG1 at different interval time (insert photograph image represent the RhB dye aspect at different time) and (**C**) Illustrates the corresponding plots of the first-order degradation rate in the presence of g-C_3_N_4_, SG1, SG2, and SG3 photocatalyst under visible-light irradiation.

The photocatalytic performance of the as-prepared catalysts was determined by RhB dye degradation under visible light irradiation using a 40 W lamp (Sylvania, Germany) as the light source. Figure 10a depicts the photo-catalytic degradation of RhB with different photocatalyst with the same weight of catalysts. As illustrated, the Ag/AgBr content in heterostructures affected significantly on their photocatalytic performance. Briefly, the degradation efficiencies of RhB are 32.88%, 64%, 82.85%, 82.84% and 100% for pure g-C_3_N_4_, Ag/AgBr, SG3, SG2, and SG1 after irradiation for 120 min, respectively. It’s clear that the SG1 of higher of the higher absorbent nature and optical conductivity σ_opt_ possessed higher photocatalytic activity. Furthermore, the Fig. 9b shows the absorption spectral changes of RhB by SG1 at different interval time.

The linear relationship of ln(C_0_/C) against time is displayed in Fig. 10c. The corresponding kinetic rate constant (k) and regression coefficients (R^2^) were estimated and listed in Table 3. According to the obtained results, the degradation reaction followed first-order kinetics. The apparent rate constants of g-C_3_N_4_, SG1, SG2, SG3, and Ag/AgBr are 0.00448 ± 3.83772E^-4^ min^−1^, 0.04531 ± 0.0071 min^−1^, 0.01704 ± 0.00365 min^−1^, 0.01132 ± 0.00268 min^−1^ and 0.00823 ± 6.62489E-4 min^−1^, respectively. It’s clear that, the rate constant of SG1 is the highest compared to other catalysts, in which the photocatalytic activity enhanced approximately 10.1-fold compared to pure g-C_3_N_4_ and 5.5-fold compared to Ag/AgBr.

**Table 3:** The corresponding kinetic constant (k) and regression coefficients (R2) of g-C_3_N_4_, SG1, SG2, SG3, and Ag/AgBr.

Sample	Kinetic constant k, min^−1^	R^2^
g-C_3_N_4_	0.00448 ± 3.83772E^-4^	0.95
SG 1	0.04531 ± 0.0071	0.93
SG 2	0.01704 ± 0.00365	0.84492
SG 3	0.01132 ± 0.00268	0.81656

### Characterization and photocatalytic activity of PDMS − Ag/AgBr@g-C_3_N_4_ sponge

**Figure 11:**
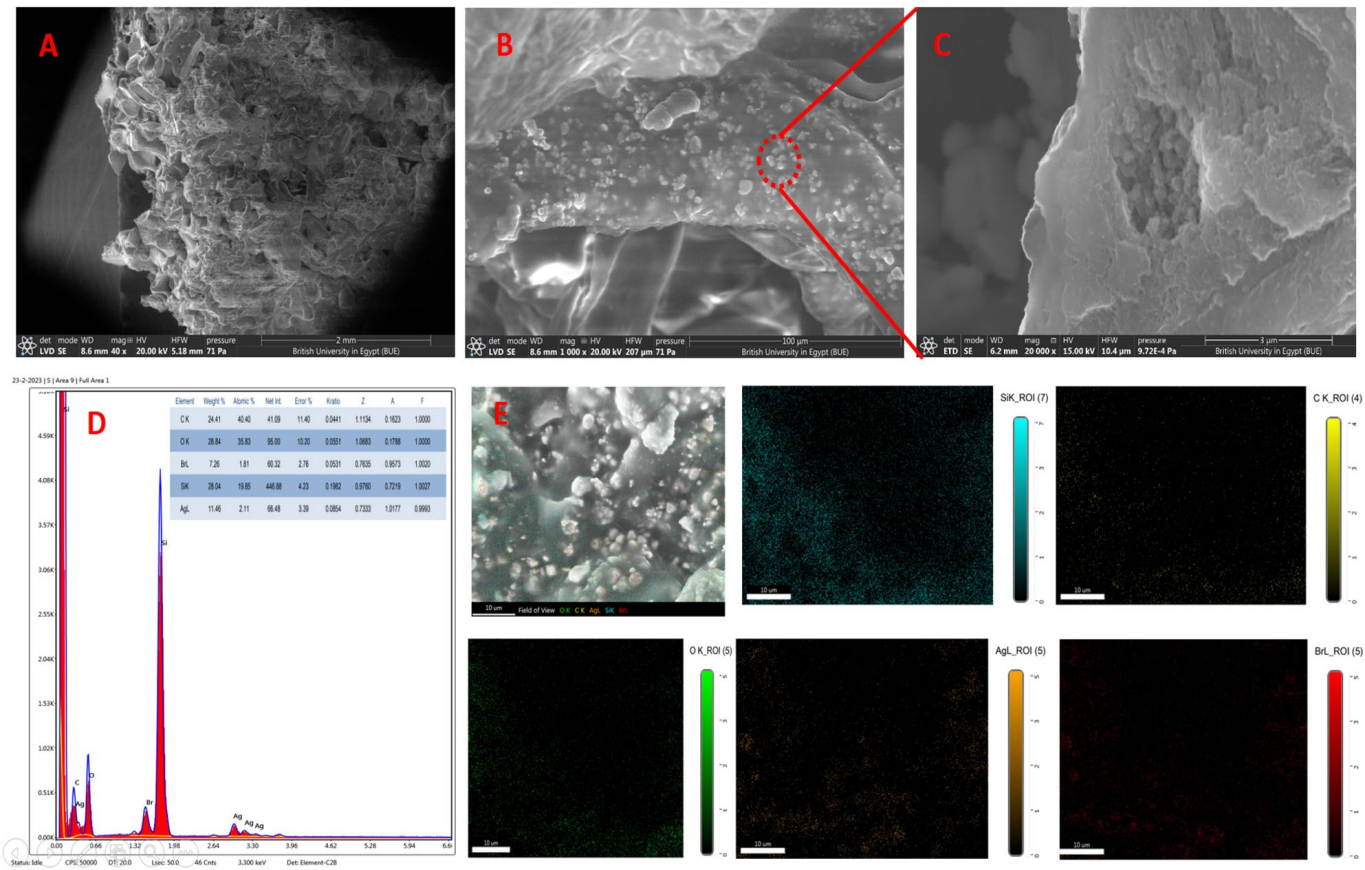
Depicts SEM images (**a**, **b** and **c**) and corresponding EDAX spectrum of the cross section of the PDMS − Ag/AgBr@g-C_3_N_4_ sponge (**d**). Elemental mapping of detected elements (**e**).

The morphology and elemental analysis of the sponge were investigated via SEM, EDAX and elemental mapping. Figure 11. depicts SEM images and corresponding EDAX spectrum of the cross section of the PDMS − Ag/AgBr@g-C_3_N_4_ sponge. The SEM images revealed that the pore structure of PDMS matrix and embedding of Ag/AgBr@g-C_3_N_4_ in the pore interface of PDMS. Elemental composition of the prepared PDMS − Ag/AgBr@g-C_3_N_4_ sponge was determined by EDAX analysis in Fig. 10d sponge composed with C, Ag, Br, O, and Si indicated pure intercalation of Ag/AgBr@g-C_3_N_4_ into PDMS matrix furthermore, elemental mapping shows homogenous distribution of Ag/AgBr@g-C_3_N_4_ photocatalyst in PDMS matrix.

**Figure 12:**
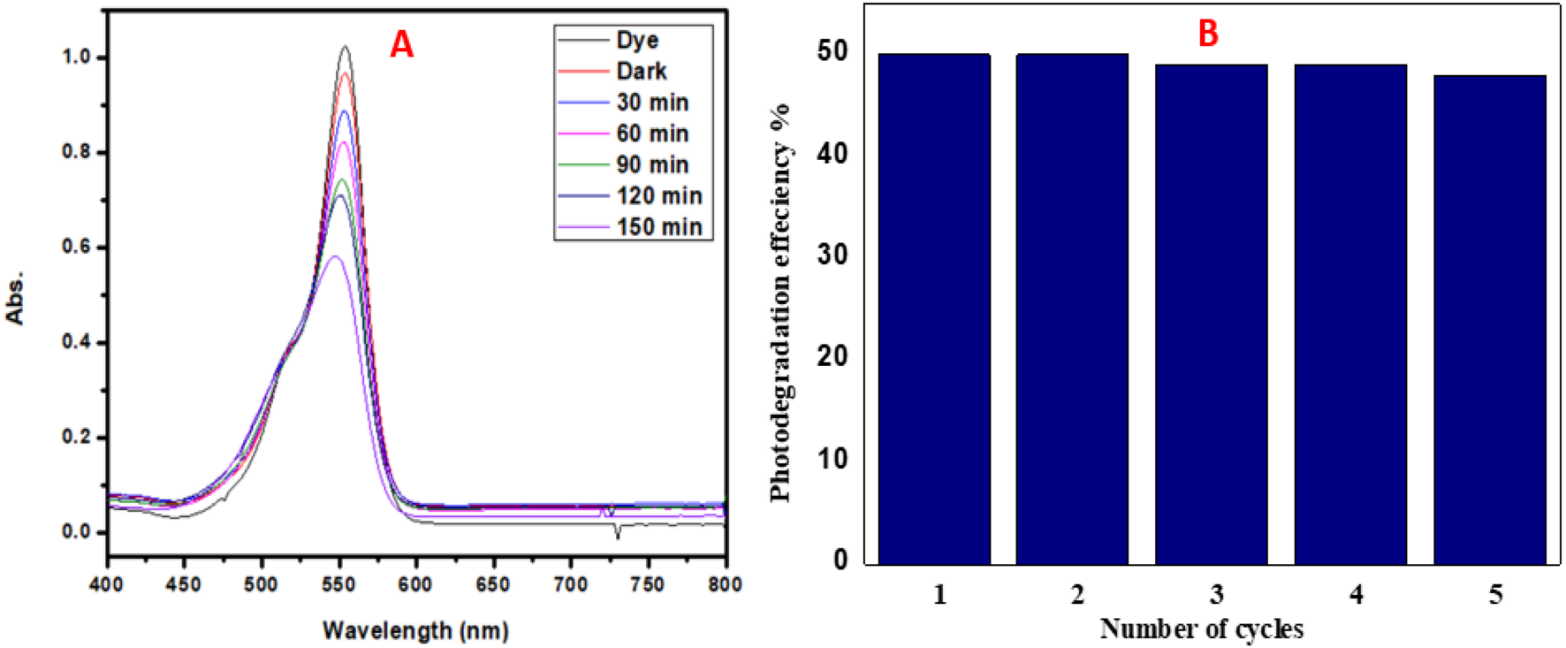
Signifying the stability and the durability of the as-fabricated sponge*.*

As illustrated in Fig. 12 the RB concentration decreased under visible light with using PDMS − SG1 sponge, reached to 50% decomposition after 150 min However, its efficiency is low comparing to the powder photocatalyst, it possesses almost the same efficiency for 5 consecutive cycles. Furthermore, the recyclability test results demonstrate that the PDMS-SG1 sponge can maintain its photocatalytic activity even after multiple reuses. The sponge exhibited stable performance for at least five consecutive runs, indicating its potential for prolonged utilization. This observation further supports the strong adhesion of the Ag/AgBr@g-C_3_N_4_ photocatalyst to the surface of PDMS pores, as previously reported^41^. These findings validate the effectiveness and consistency of the developed sponge-based photocatalytic system, aligning with previous research in the field.

**Figure 13:**
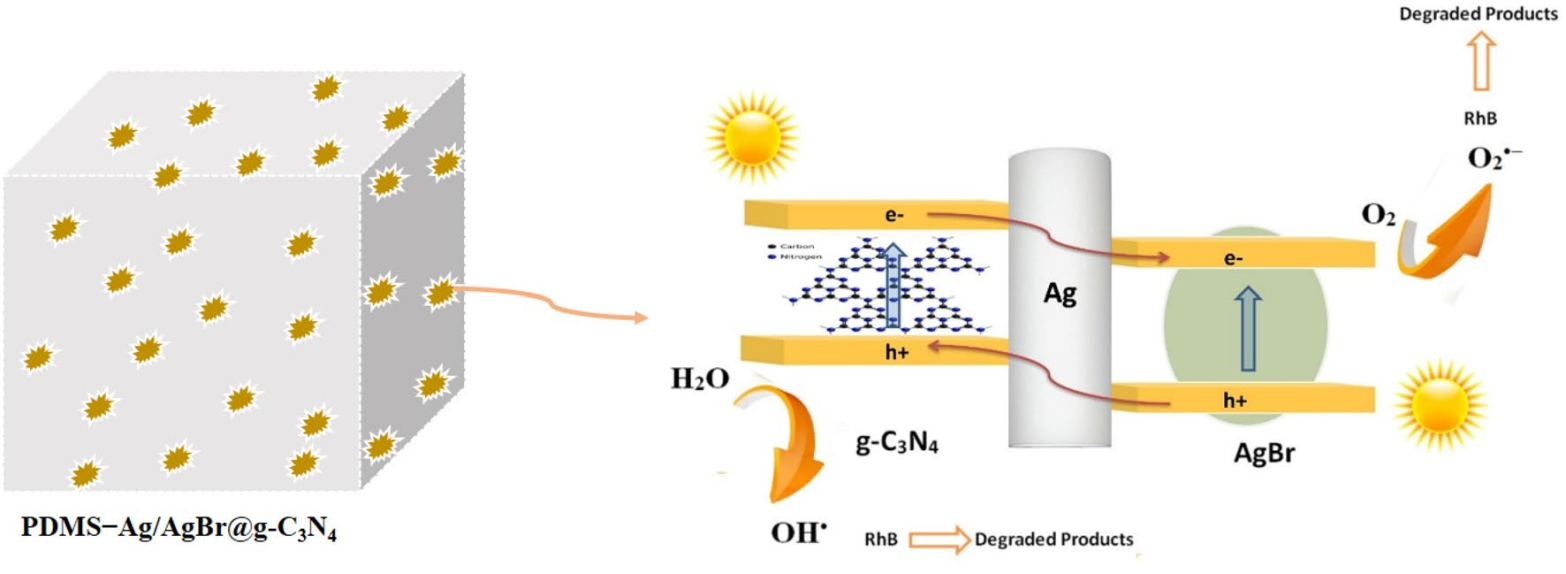
The suggested mechanism of the degradation process over Ag/Ag@g-C_3_N_4_ nanocomposite.

The mechanism of degradation reaction is suggested based on the above results and previous work in Fig. 13. The high adsorption capacity of porous structures enables organic pollutants to be effectively located near the Ag/AgBr@g-C_3_N_4_ attached to the pore surface then, both g-C_3_N_4_ and AgBr incorporated onto PDMS sponge were excited by visible-light. After that, (I) photogenerated charges are formed on the g-C_3_N_4_ and AgBr surface simultaneously then, the excited electrons of g-C_3_N_4_ transfer to the CB of AgBr and leave the positively charged g-C_3_N_4_^n+^. (II) The electron–hole pairs in AgBr are separated else by migration of h^+^ to VB of g-C_3_N_4_. concurrently, Ag NPs can be triggered by visible light due to the SPR effect, which facilitates the electron–hole separation^68^. The adsorbent molecular oxygen can react with the separated electrons to produce highly reactive radical species. Meanwhile, the h^+^ generate hydroxyl free radicals subsequently, the generated ROSs degrade the RhB pollutant.

## Conclusions

The composition between Ag/AgBr and g-C_3_N_4_ was successfully prepared, with study the impact of g-C_3_N_4_ on the structural, morphological, optical properties, chemical composition and Photocatalytic of Ag/AgBr. According to the obtained results, the particle size of Ag/AgBr was reduced upon addition of g-C_3_N_4_ while, the microstrain and dislocation density were increased. Furthermore, the addition of Ag/AgBr to g-C_3_N_4_ improve the electron–hole separation and leads to a shift of the absorption edge towards lower values of band-gap, form 2.646 eV (pure g-C_3_N_4_) to 2.393 eV (SG1) resulting in improvement in extinction coefficient k, optical conductivity σ_opt_, and small shift and augmentee in the ε′ and ε″ values hence, the photocatalytic activity was enhanced. Finally, this work provides an intensive study to elucidate the relationship between the structural, optical parameters and photocatalytic activity, to prepare efficient Ag/AgBr@g-C_3_N_4_ photocatalyst, after that immobilized on a floating, porous PDMS sponge surface via a facile sugar-template technique. The sponge showed lower photocatalytic efficiency than the pure catalyst under visible light but the sponge is stable enough to be reused several times. Based on its simple fabrication, photocatalytic activity and high recyclability, the PDMS − Ag/AgBr@g-C_3_N_4_ sponge is a promising material for environmental applications and energy conversion purposes and allows the reuse of the photocatalyst several times. Further studies will be done to overcome the PDMS barrier between photocatalyst and surrounding medium to improve its photocatalytic performance. Finally, this work offered a promising and feasible technology for efficient removal of organic pollutants from the environment and generating clean energy.

## Data Availability

The datasets used and/or analysed during the current study available from the corresponding author on reasonable request.
